# Exploring the changing geographical pattern of international scientific collaborations through the prism of cities

**DOI:** 10.1371/journal.pone.0242468

**Published:** 2020-11-16

**Authors:** György Csomós, Zsófia Viktória Vida, Balázs Lengyel

**Affiliations:** 1 Department of Civil Engineering, University of Debrecen, Debrecen, Hungary; 2 Department of Science Policy and Scientometrics, Library and Information Centre of the Hungarian Academy of Sciences, Budapest, Hungary; 3 Agglomeration and Social Networks Lendület Research Group, Centre for Economic- and Regional Studies, Budapest, Hungary; 4 Centre for Advanced Studies, Corvinus University of Budapest, Budapest, Hungary; Max Planck Society, GERMANY

## Abstract

Science is becoming increasingly international in terms of breaking down walls in its pursuit of high impact. Despite geographical location and distance still being major barriers for scientific collaboration, little is known about whether high-impact collaborations are similarly constrained by geography compared to collaborations of average impact. To address this question, we analyze Web of Science (WoS) data on international collaboration between global leader cities in science production. We report an increasing intensity of international city-city collaboration and find that average distance of collaboration of the strongest connections has slightly increased, but distance decay has remained stable over the last three decades. However, high-impact collaborations span large distances by following similar distance decay. This finding suggests that a larger geographical reach of research collaboration should be aimed for to support high-impact science. The creation of the European Research Area (ERA) represents an effective action that has deepened intracontinental research collaborations and the position of the European Union (EU) in global science. Yet, our results provide new evidence that global scientific leaders are not sufficiently collaborative in carrying out their big science projects.

## 1. Introduction

The decrease of communication and travel costs since the 1990s has enabled interactions between distant partners. However, and despite the early visions developed in the geography literature on the decreasing significance of distance [1–6], it is repeatedly found that the majority of social interactions are spatially bounded [7–9]. Research is no exception: the probability of collaborations decreases as distance grows, as has been found for co-authorship relations [10–12], EU-supported research collaboration [13], and inventor collaboration [14]. What is not entirely clear yet is how the quality of collaborative output influences the distance-dependence of scientific collaboration. Are high-impact collaborations similarly constrained by distance in the same manner as for collaborations of lower impact?

High-impact research—reflected by the number of citations a publication receives—is increasingly a multi-university phenomenon [15], in which the combination of diverse knowledge located in many departments pays off in better-received publications. However, it is still debated whether spatial concentration or spatial diversity produces more high-impact papers. Jones et al. [15] claim that high-impact publications concentrate in spatially concentrated elite universities. In this line, Abbassi and Jaafari [16] find that national collaboration favors citations more than international collaboration. On the contrary, scientific collaboration in Europe has been found to have higher impact when collaborators are from many countries [17, 18].

International collaboration in science is gaining importance [12, 19, 20] and is further supported by research funders, primarily in the European Union [21, 22]. In recent decades, international scientific collaboration in terms of the number of co-authored papers has experienced a remarkable growth rate [23]. For example, Wagner et al. [24] found that, between 1990 and 2011, the ratio of internationally co-authored records in the SCI dataset increased from 10 to 25 percent. It is well studied that international collaboration is not only highly beneficial for participants [25, 26] but in some cases (e.g., for “big science”) it is essential [27–29]. In addition, those publications that were produced in international research projects generally received more citations [30, 31]. It is, however, important to notice that according to research conducted by Maisonobe et al. [32], in many countries, domestic collaborations increased faster than international collaborations.

In this paper, we focus on international co-publication links between global leader cities in science production and evaluate the changing role of distance over the last three decades by analyzing Web of Science (WoS) data. To varying degrees, cities are major sites of science production in terms of the number of publications and citations. The question regarding how cities participate in global science is extensively analyzed in the growing field of spatial scientometrics [33–52].

The pioneer work of spatial analysis focusing on the city level was produced by Matthiessen and Schwarz [33], who examined the scientific strength in terms of publication output of “greater” urban regions of Europe. Since the beginning of the 2010s, this rather quantitative approach has been replaced by a new paradigm focusing on the geographical context of the production of research excellence. Bornmann et al. [34] and Bornmann and Leydesdorff [35, 36] identified and mapped cities that were considered to be centers of excellence in scientific research on the basis of the size and frequency of the production of top 1% highly cited papers. Bornmann and de Moya-Anegón [37] mapped German cities, with most papers belonging to the 1% most frequently cited papers, within their subject area and publication year. Bornmann and de Moya-Anegón [38] detected hot and cold spots in the United States based on bibliometric data produced by institutions. Other researchers investigated some additional aspects of cities’ participation in science. Grossetti et al. [39] examined the global and national deconcentration of scientific activities through the lens of cities. Csomós and Tóth [40] investigated the spatial distribution of scientific publications produced by the industry. Csomós [41] examined the publication dynamics, collaboration pattern, and disciplinary profile of more than 2,000 cities worldwide, and in another contribution, Csomós [42] revealed factors that may influence cities’ high impact efficiency. Leydesdorff and Persson [43] displayed co-authorship, collaboration networks between cities by using mapping and network visualization software. The article produced by Wu [44] proposed a citation rank based on spatial diversity in terms of cities and countries, focusing on the measurement of the spatial aspect in citation networks. Jiang et al. [45] investigated the spatial patterns of R&D collaborations of Chinese cities by using co-patent data, and Andersson et al. [46] revealed the internal spatial structure of China’s scientific output. Catini et al. [47] explored spatially concentrated innovation clusters within metropolitan areas by geocoding publication data. Maisonobe et al. [32, 48–50] investigated cities’ publication output and collaboration network from different aspects. They found “that cities located in scientific emerging countries tended to favor domestic interurban co-authorships whereas cities located in more traditionally English-speaking countries internationalized” [32].

These papers cover varying topics in the domain of spatial scientometrics, focusing on the city level.

However, scientific interaction between cities has been analyzed by only a few papers [32, 53, 54], primarily due to the problems of data collection and processing [55].

We make several new contributions to this literature. We document that the intensity of inter-national collaborations of cities is gradually growing over time. In the meantime, we observe a shift in the average geographical distance that occurs for both low- and high-intensity city−city collaborations as well. However, this shift leaves distance decay unchanged: the average distance of collaborations decreases monotonically as the intensity of collaborations between cities increases, and the pattern is stable over time. Most importantly, we find that a similar distance decay spans over larger distances for high-impact collaborations, meaning that the most important global collaborations require large geographical coverage. Results show that cities located in the European Union construct the most intense international research collaboration. Since the mid-2000s, the creation of the European Research Area (ERA) and the enlargements of the Community have given significant impetus to the deepening of intracontinental research collaborations. However, major scientific actors, that is the United States, the European Union, and Japan, tend to carry out big science projects separately from each other.

## 2. Materials and methods

### 2.1 Data

We have collected a number of co-authored publications between the top 245 global science producer cities for the periods 1994−1996, 2004−2006, and 2014−2016. That is, we capture scientific cooperation between two cities if a publication is produced by at least two authors located in those cities; and it is international if those cities are located in different countries [26, 56, 57]. We also analyzed the geography of scientific collaboration in highly cited papers (HCPs). HCPs are those papers that receive sufficient citations to belong to the top 1% of their academic fields, taking the most recent 10-year period into account.

To conduct the bibliometric analysis, the WoS database was employed, which is considered to be one of the most prestigious abstract and citation databases [58–60] and is widely used for carrying out spatial analysis [see, for example, 20, 61–67]. The WoS provides four major indexing databases for journal articles (SCIE, SSCI, A&HCI, and ESCI), out of which the SCIE (Science Citation Index-Expanded) and SSCI (Social Sciences Citation Index) were employed. The SCIE and SSCI together list more than 12,800 journals and cover such broader areas as life sciences and biomedicine, physical sciences, technology, and social sciences.

We consider only those cities where at least 10,000 articles were published during 2014−2016. The publication history of these selected cities was investigated in the periods of 1994−1996 and 2004−2006 as well. Naturally, one of the major problems of spatial scientometric analysis focusing on the city level is that it is rather challenging to delineate cities according to the same spatial standards [55]. For example, in urban geography, the name “Tokyo” can correspond to both the Tokyo Metropolis with an area of 2,200 square kilometers and a population of 14 million and the Tokyo Major Metropolitan Area covering 32,700 square kilometers and containing 36.3 million people (i.e., they may produce highly different publication outputs). To remain consistent, in our analysis, the “city” corresponds to the spatial unit that is reported by the author(s) in the affiliation field of the article, being placed between the name of the country (state/prefecture/etc.), and that of the organization the author(s) are affiliated with. Table 1 demonstrates the distribution of selected cities across macro-regions (in addition, a more thorough explanation on the topic can be found in Section 3.3). As can be seen, more than two thirds of the cities being involved in this analysis are located in Western Europe and Northern America (with a dominance of the United States). Based on the number of cities, Asia (with the major proportion of Chinese and Japanese cities) comes third. The contribution of the European Union to the total number of cities is 37.5 percent.

**Table 1:** Summary statistics of cities involved in the analysis.

Macro-region	Number of cities	Percentage of cities in the dataset	Number of papers, 1994–1996	Number of papers, 2004–2006	Number of papers, 2014–2016	Number of HCPs, 2014–2016
Africa	2	0.816	5,601	6,942	21,914	471
Asia	44	17.959	251,565	628,930	1,596,164	19,571
Australia	8	3.265	42,625	83,144	208,525	3,914
Eastern Europe	9	3.673	92,549	119,813	200,878	2,100
Latin America	7	2.857	28,251	74,296	160,619	1,598
Middle East	9	3.673	35,711	75,644	198,067	2,236
Northern America	74	30.204	581,229	1,273,062	1,967,595	45,110
Western Europe	92	37.551	791,967	1,223,389	2,145,969	43,846
**Total**	**245**	**100.000**	**1,829,498**	**3,485,220**	**6,499,731**	**118,846**
European Union*	*92*	*37*.*551*	*778*,*285*	*1*,*211*,*981*	*2*,*125*,*969*	*41*,*862*

* In the case of the European Union, the community of 28 member states is considered, irrespective of which of the periods is examined

The dataset that demonstrates the geographical classification and publication outputs of cities, as well as the number of co-authored papers and the Jaccard indexes of the top 3,000 collaboration links by each period, is available at Harvard Dataverse (https://doi.org/10.7910/DVN/WRGHHT). The dataset regarding the disciplinary breakdown of publications produced by cities is also available at this site.

### 2.2 Methods

When investigating the international scientific collaboration trends of cities, a threshold was set in the case of each period. Theoretically, the collaboration matrix of cities contains 29,890 [(n × (n-1))/2, where n = 245] links, out of which the maximum number of international collaboration links is 26,990. However, most city-dyads produced a rather weak collaboration in terms of the number of co-produced articles. In addition, primarily in the first period (1994−1996), but also in the second period (2004−2006), many cities did not maintain international collaboration, and they only collaborated with their domestic peers. Therefore, it was reasonable to establish minimum collaboration values regarding each period between city-dyads, which are as follows: 1994−1996: 10 co-produced articles per year; 2004−2006: 30 co-produced articles per year; and 2014−2016: 90 co-produced articles per year. When choosing the above threshold values, the increase in the world’s publication output was considered [68]. Based on these thresholds, cities produced a total number of 3,122, and 3,111 collaboration links in the periods of 1994−1996, and 2004−2006, respectively. In the most recent period, however, the total number of collaboration links increased to 7,827, regardless of the fact that the threshold was set high. In each period, based on their relative strength, the top 3,000 collaboration links were considered. Naturally, it is highly likely that if two cities, irrespective of where they are located, produce high publication outputs, they will build stronger collaboration in terms of the number of co-authored publications, compared to cities with smaller publication outputs. To reduce the size effect and determine the relative strength of a particular collaboration link, the Jaccard similarity index was employed [56]:

$$J_{x,y}=\frac{C_{x,y}}{\left(C_{x}+C_{y}-C_{x,y}\right)}$$ (1)

where *J*_*x*,*y*_ is the relative strength of a given collaboration link, *C*_*x*,*y*_ is the number of co-produced publications of cities *x* and *y*, and *C*_*x*_ and *C*_*y*_ are the total publication outputs of city *x* and *y*, respectively.

The main reason for applying the Jaccard index for analyzing the role of distance is that we do not have access to the number of scientists located in the cities we analyze. Therefore, we cannot turn to conventional gravity models and compare the observed volume of collaboration with the potential number of collaborations [9] or with the expected number of collaborations retrieved from regression estimation [14]. Instead, the Jaccard-like measures, in which the strength of nodes are used to scale down the dyad weight, have been shown to produce distance-decay patterns [8] and are therefore appropriate for our problem.

Let us take the example of the Boston−London pair to illustrate the relation between the Jaccard coefficient and the raw number of collaborations. In the period of 2014−2016, there were 4,735 co-authored publications including authors from Boston and London, which is the second-highest number of collaborations. In that period, with 153,725 publications, London’s output was the second largest in the world, and Boston was ranked fifth with 105,769 publications. After calculating the relative strength of that collaboration of the city-dyad (*J*_*x*,*y*_ = 0.018586), it turned out that it was occupying only 207^th^ place in the ranking.

We consider it important to demonstrate how high-impact research collaborations relate to geographical distance, and whether geographical proximity affects the intensity of those collaborations. In the past few years, a number of studies have been published focusing on identifying and ranking the centers of excellence across the world [see, e.g., 34, 35, 69–71]. In those works, the number and/or ratio of highly cited papers are employed as a proxy to express research excellence. In the case of the period of 2014−2016, we compared the relative strengths of international scientific collaborations based on the Jaccard indexes derived from all papers, and HCPs exclusively.

By employing this method, it was possible to compare how international scientific collaboration between cities had developed over time. In addition, in the case of the period of 2014−2016, it was also examined how cities participate in the production of highly cited papers; that is, which of the collaboration links were considered the relatively strongest when producing excellent papers. Finally, each collaboration link was mapped to explore the changes in the geographical pattern of those collaborations.

## 3. Results

### 3.1 Distance and intensity of international scientific collaboration between cities

**Fig 1:**
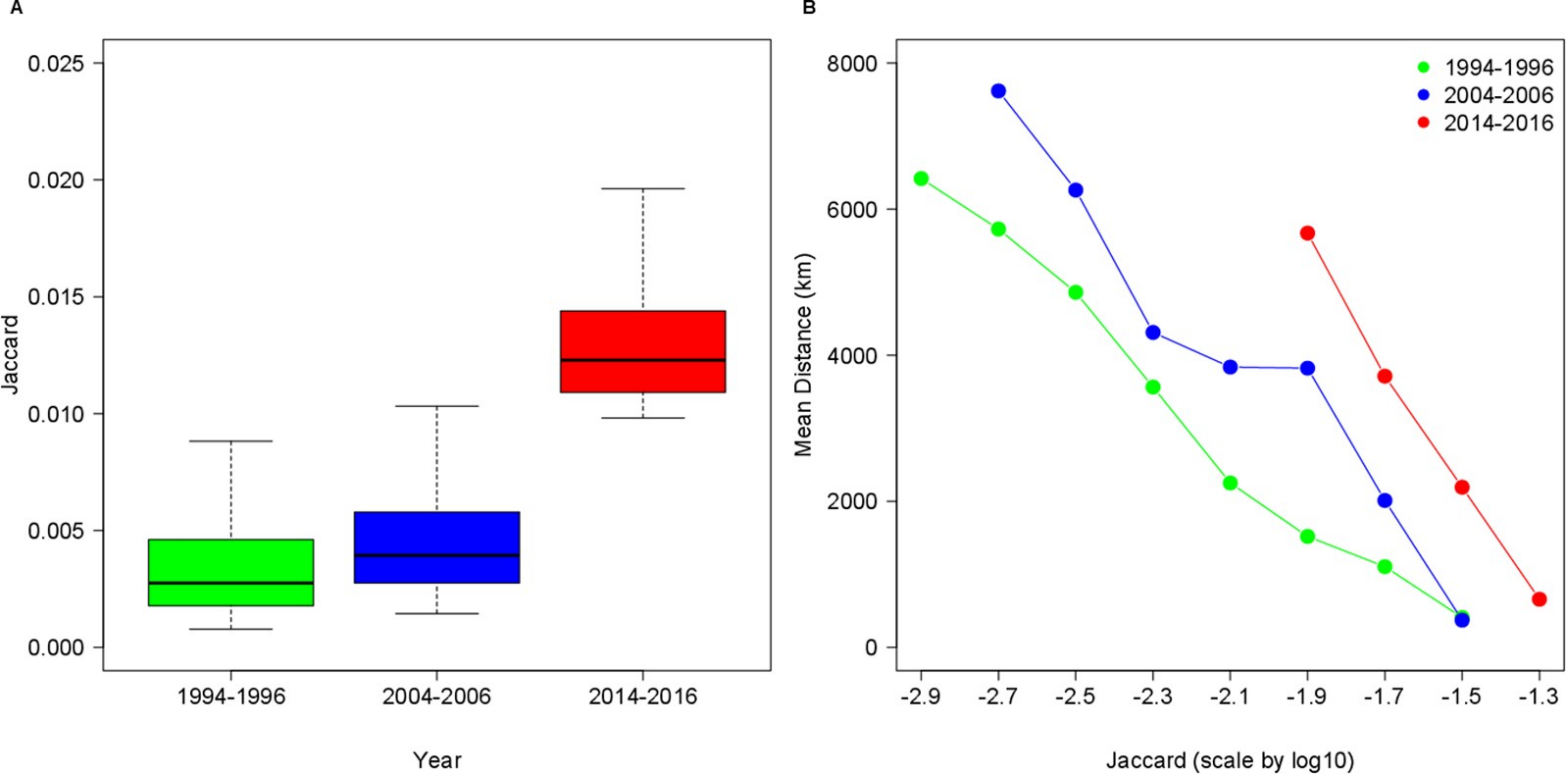
Intensity and distance decay of international scientific collaboration between cities. **A.** Changing intensity of international scientific collaboration between cities is reflected by the distribution of the Jaccard index. **B.** Changing mean distance of top 3,000 collaboration links between cities in the periods of 1994−1996, 2004−2006, and 2014−2016.

The intensity of international collaboration between cities, measured by the Jaccard index, has witnessed a rather small change from 1994−1996 (*μ*_*J*_ = 0.003547) to 2004–2006 (*μ*_*J*_ = 0.004599), but the magnitude of the increase was observed by 2014−2016 (*μ*_*J*_ = 0.013212). These results illustrated in Fig 1A suggest that besides the previously reported general rise of international scientific collaboration [23], the pairwise intensity of city-city collaboration has increased since the mid-2000s. In other words, not only the magnitude of international collaboration has risen, but also its intensity of collaboration already controlled for the size effect of cities.

The geographical reach of intensifying international collaboration has widened, while distance decay remained an important factor of collaboration intensity between two cities. Fig 1B demonstrates that distance decay curves have shifted up and to the right as well over the decades. For example, the mean distance of the weakest collaboration links at a 10^−2.7^ Jaccard value had covered approximately 6,000 kilometers on average in 1994−1996, which almost increased to 8,000 kilometers by 2004−2006. At the same time, the general increase of Jaccard shifts the decay curves to the right: the smallest value of Jaccard 10^−1.9^ in 2014−2016, for which the average distance is around 6,000 kilometers. These observations mean that recently much stronger collaborations (in terms of the number of co-produced papers scaled down by city production) have been established between cities even if they are located at an increased distance from each other. Yet, taking each time period, even the latest one into account, the mean distance curves are sloping downwards from the lowest Jaccard index category to the highest one. This finding implies that those cities that are located a further distance from each other, particularly if they are located on different continents, establish relatively less intense scientific cooperation in the given period. The stable patterns of distance decay are due to the dominance of European collaborations with other European and Northern American cities (on this issue, see a more thorough explanation in Section 3.3).

**Fig 2:**
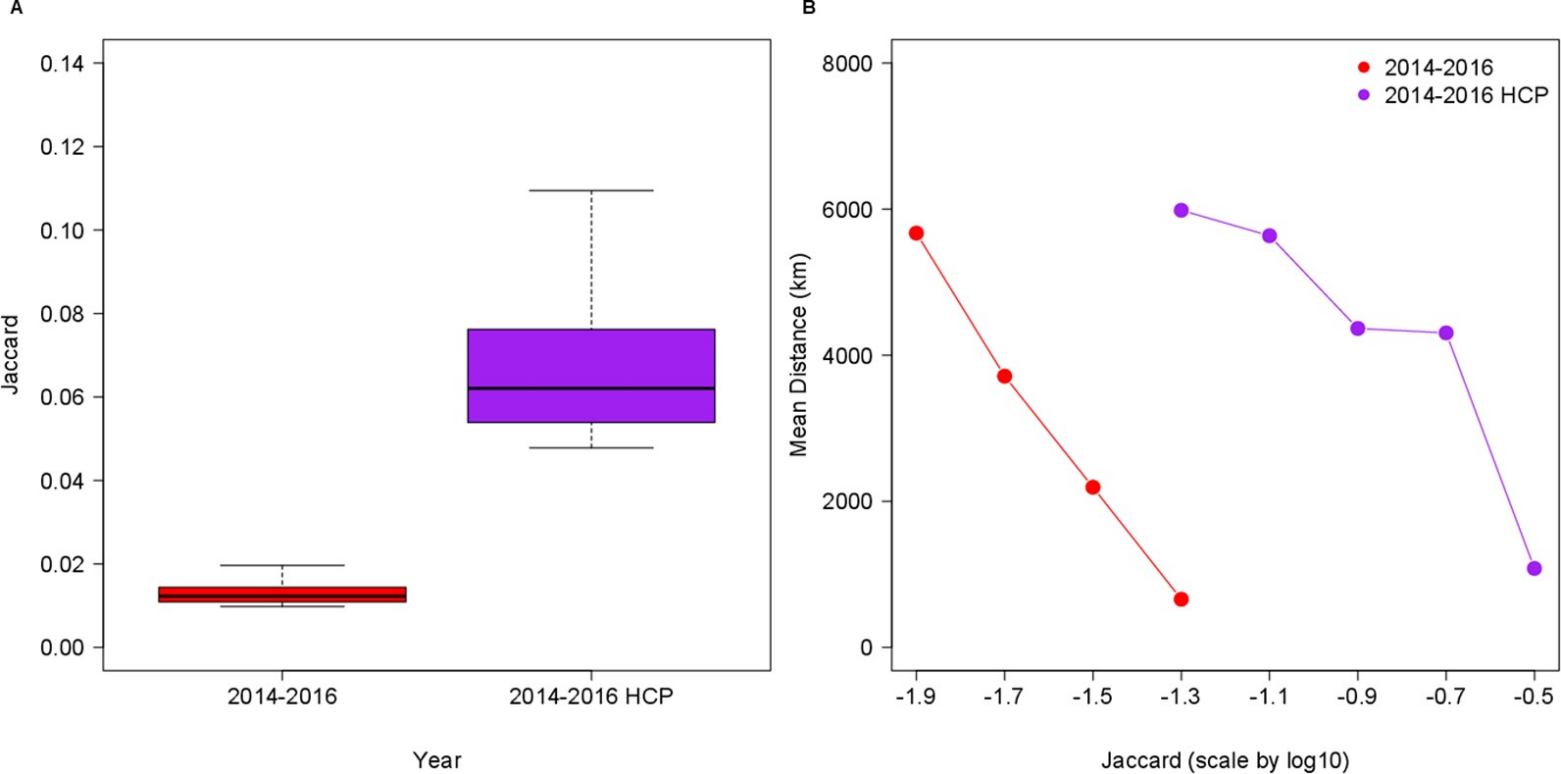
Comparing intensity of international scientific collaborations between cities in the cases of all papers and HCPs exclusively. **A.** International collaboration intensity is higher for HCP than for general papers. **B.** Mean distances of the top 3,000 collaboration links if all papers and HCPs are considered, respectively.

In Fig 2, we compare the collaboration intensity of publications that belong to the top 1% based on the number of citations they received with the general collaboration patterns in the 2014−2016 period. As can be seen in Fig 2A, the distribution of inter-city collaboration intensity is much higher in the case of HCP production. In the period of 2014−2016, the mean Jaccard index of the top 3,000 collaboration links producing HCPs (*μ*_*J*_ = 0.068663) was more than five times higher than that of the top 3,000 collaboration links. These findings suggest that the production of HCPs that are deemed to be the outcomes of large-scale research projects requires deeper cooperation from international actors.

In addition, as demonstrated in Fig 2B, in the case of HCPs, international collaborations between cities are less dependent on the effect of geographical proximity. The mean-distance curve of the HCP collaborations starts from an approximately same mean distance level but at a higher Jaccard index category than the general curve. Hence, in the case of HCPs, stronger international collaborations are created between cities at distances that allow for relatively weak collaborations otherwise. Naturally, geographical proximity still matters in the case of HCP collaborations; the curve of those collaborations follows a similar slope.

### 3.2 Disciplinary profiles of high scientific impact

Citation trends differ across disciplines. Therefore, we examine the distribution of papers across major scientific fields considering the difference between average- and high-impact papers and examine the disciplinary profiles of cities. This approach will enable us to better explain what is behind the continental distribution of high-impact collaboration in the next section.

**Fig 3:**
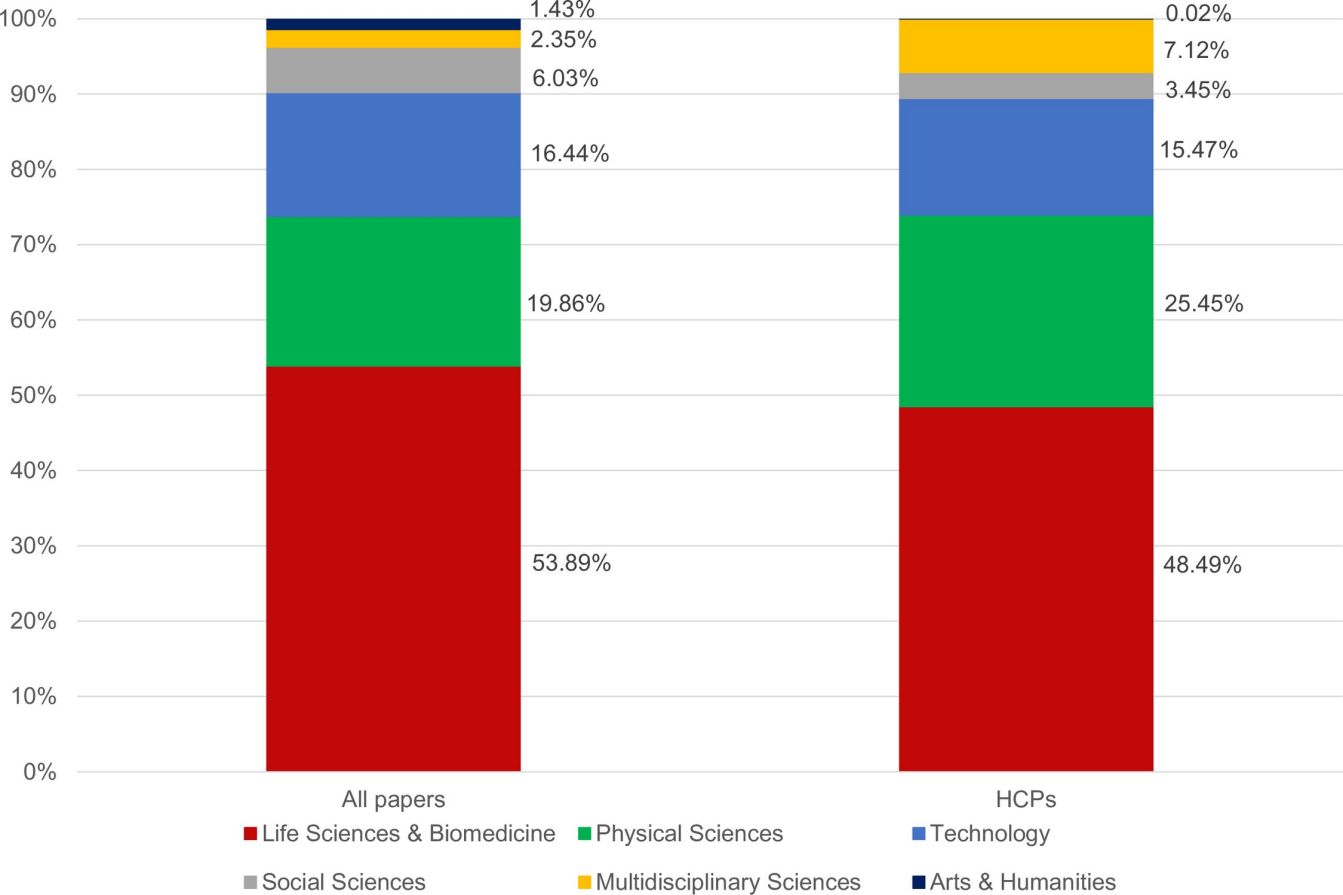
Disciplinary profile of selected cities’ publication outputs. **A.** For all papers. **B.** For papers with top 1% citations.

Fig 3 illustrates that taking all papers from selected cities, the largest proportion of papers are published in the fields of life sciences, physical sciences, and technology (the classification is based on the WoS Research Area classification). The contribution ratio of these fields to the total output of the 245 cities is 91 percent, with life sciences holding the dominant position. If focusing on the HCP outputs, the fields of life sciences, physical sciences, and technology produce almost the same contribution ratio, but their internal ratios have changed: fewer HCPs are published in the field of life sciences, whereas the field of physical sciences produces more HCPs. In addition, the contribution ratio of social sciences is much smaller in the case of HCPs, whereas that of multidisciplinary sciences has increased.

In the WoS, the research papers published in multidisciplinary journals, such as Nature, Science, PNAS, and PLoS ONE, are classified as multidisciplinary, irrespective of their exact disciplinary profiles. Approximately 47–48 percent of the research papers published in Nature and Science can be classified in the field of life sciences and 21–22 percent of them belong to the fields of physics and chemistry (i.e., physical sciences); in contrast, nearly 95 percent of the research papers in PNAS are published in the field of life sciences [72]. In sum, the growing share of multidisciplinary sciences among HCPs intensifies the overrepresentation of life sciences and physical sciences.

Examining the breakdown of broader disciplinary categories enables us to realize that the majority of top disciplines in terms of the number and share of HCPs belong to the fields of life sciences, physical sciences, and technology (Table 2). These disciplines combined with multidisciplinary sciences (i.e., 20 out of the 233 disciplines) provide more than 50 percent of HCPs that have been produced in the selected cities over 2014−2016.

**Table 2:** Disciplinary classification of HCPs produced by cities.

Disciplines	Broader discipline categories	Total number of HCPs produced in 2014−2016	Ratio of HCPs (%)	Number of cities in which particular HCPs are produced
Multidisciplinary Sciences		13,214	7.119	245
General Internal Medicine	Life Sciences	12,037	6.485	243
Oncology	Life Sciences	8,203	4.419	241
Chemistry, Multidisciplinary	Physical Sciences	7,402	3.988	237
Physics, Particles & Fields	Physical Sciences	7,055	3.801	214
Astronomy & Astrophysics	Physical Sciences	6,501	3.502	222
Materials Science, Multidisciplinary	Technology	5,240	2.823	232
Environmental Sciences	Life Sciences	4,128	2.224	239
Physics, Multidisciplinary	Physical Sciences	4,116	2.217	231
Chemistry, Physical	Physical Sciences	4,058	2.186	224
Cardiac & Cardiovascular Systems	Life Sciences	4,053	2.183	225
Physics, Applied	Physical Sciences	3,695	1.991	225
Nanoscience & Nanotechnology	Technology	3,645	1.964	217
Biochemistry & Molecular Biology	Life Sciences	3,536	1.905	236
Cell Biology	Life Sciences	3,162	1.703	239
Neurosciences	Life Sciences	3,010	1.622	224
Public Environmental Occupational Health	Life Sciences	2,956	1.593	224
Engineering, Electrical Electronic	Technology	2,554	1.376	197
Physics, Condensed Matter	Physical Sciences	2,533	1.365	207
Energy Fuels	Technology	2,455	1.323	216
Other Disciplines		82,067	44.212	
Total (233 disciplines)		185,620	100.000	

Recently, research projects both in the case of life science disciplines, such as oncology and neuroscience, and physical science disciplines, including particle physics, astronomy, and astrophysics have been carried out by international collaborations in large-scale research teams. Some of these projects, particularly those carried out in various branches of physics are often labeled as “big science” projects because they are highly complex and expensive and require a research team of hundreds or thousands of scientists and engineers, as well as major research infrastructure, including research facilities, machines, and services [73–75]. In addition, over the past 50 years, research projects, particularly in the fields of natural sciences and life sciences but also in technology and social sciences, have been experiencing a substantial increase in terms of team size [76, 77]. A study by Larivière et al. [78] found that “collaborative research results in higher citation rates”; that is, those papers that are produced by large teams will receive more citations and are, thus, more likely to be highly cited, in contrast to those being produced by single authors or small research teams [79, 80]. The nexus between team size and citation rates is reinforced by Wu et al. [81], who assert that ten-person teams are 50% more likely to score a high-impact paper than those produced by solo authors and small research teams.

Big science and many large-scale research projects are typically carried out in international collaborations. For example, the Manhattan Project (1942−1946), which is generally accepted to be the earliest big science project [82, 83] was coordinated by the United States and supported by the United Kingdom and Canada. Following projects in the fields of particle physics, astronomy, and astrophysics using the infrastructure of such mega research facilities as the Large Hadron Collider operated by the pan-European research organization, CERN [84], the Spallation Neutron Source located in the Oak Ridge National Laboratory, Tennessee [85], and the Very Large Array of the National Radio Astronomy Observatory in New Mexico. In life sciences and biology, the Human Genome Project was the world’s largest collaborative project, taking place between 1990 and 2003 [86, 87]. This was followed by such highly complex multinational research projects as the Human Epigenome Project in the field of epigenomics [88], and the European Union’s flagship neuroscience project, the Human Brain Project, launched in 2013 [89]. In addition, there is evidence that international research collaboration has also been becoming increasingly important in the fields of medicine [90], cancer research [91, 92], and neuroscience [93].

### 3.3 Geographical patterns and global regions in city-city collaborations

Now, we turn to investigate the detailed geographical patterns of international scientific collaboration between cities and pay special attention to continental distributions. To outline the changes in the geographical pattern of international scientific collaborations between cities and investigate the patterns of high-impact collaborations, we classified each link into quarters based on the Jaccard index (Table 3). Each quarter contains 750 collaborations links.

**Table 3:** Classification of collaboration links into quarters by periods.

	1994–1996				2004–2006			
	Jaccard category quartile	Mean distance (km)	Standard deviation of distance (km)	Mean Jaccard index	Jaccard category quartile	Mean distance (km)	Standard deviation of distance (km)	Mean Jaccard index
Q4	0.000783–0.001780	6,140	3,927	0.0014	0.001450–0.002750	6,934	3,782	0.0022
Q3	0.001780–0.002760	5,017	3,644	0.0022	0.002750–0.003940	5,257	3,878	0.0033
Q2	0.002760–0.004610	3,934	3,595	0.0036	0.003940–0.005790	3,603	3,530	0.0048
Q1	0.004610–0.022800	2,149	2,624	0.0070	0.005790–0.021000	3,860	3,446	0.0081
	2014–2016				2014–2016 HCP			
	Jaccard category quartile	Mean distance (km)	Standard deviation of distance (km)	Mean Jaccard index	Jaccard category quartile	Mean distance (km)	Standard deviation of distance (km)	Mean Jaccard index
Q4	0.009820–0.010900	6,019	4,208	0.0104	0.047800–0.053900	5,832	4,446	0.0506
Q3	0.010900–0.012300	5,449	4,319	0.0116	0.053900–0.062100	5,820	4,463	0.0577
Q2	0.012300–0.014400	4,106	3,989	0.0133	0.062100–0.076200	5,431	4,492	0.0685
Q1	0.014400–0.045300	3,123	3,618	0.0176	0.076200–0.212000	4,262	4,147	0.0977

**Fig 4:**
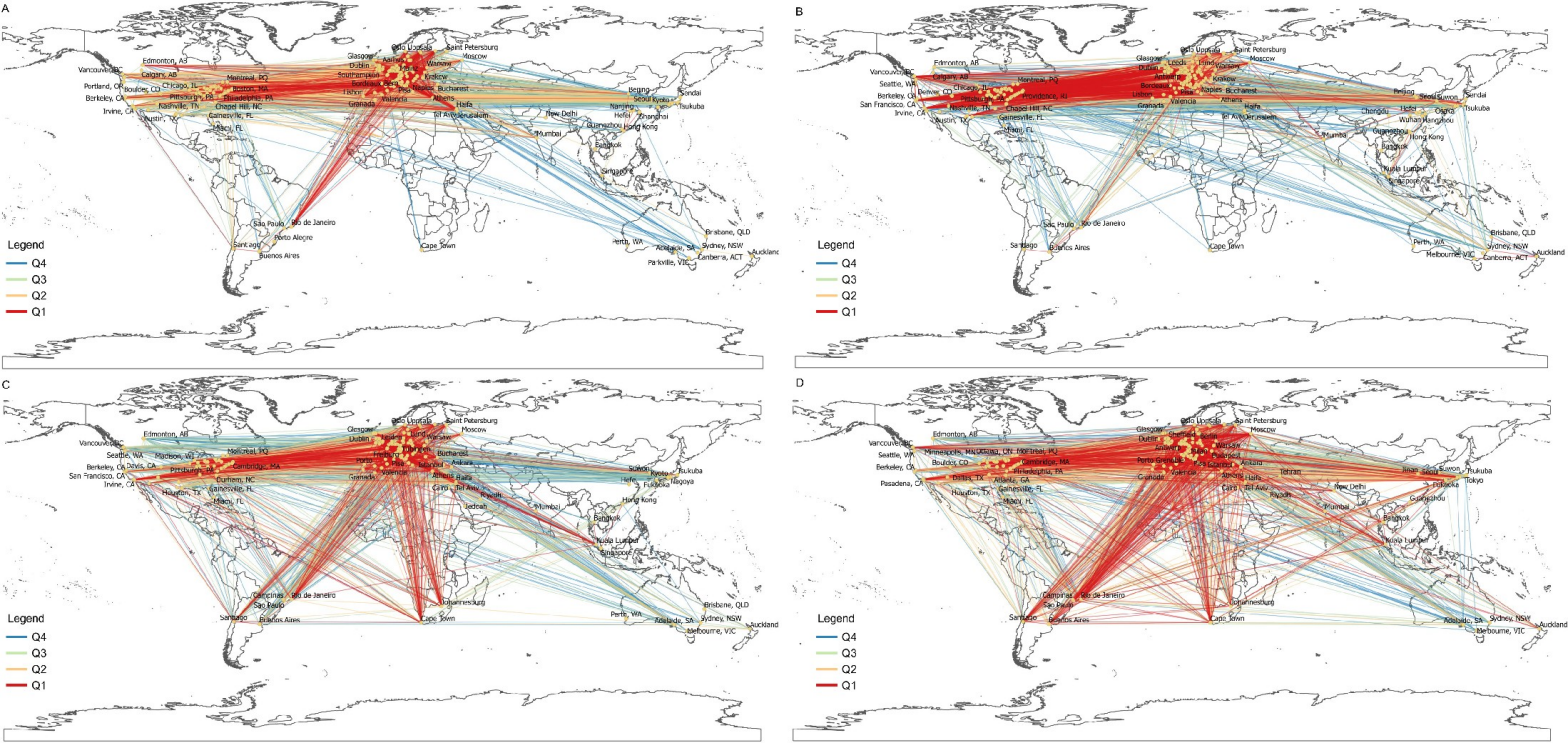
Geographical pattern of international scientific collaborations between cities based on the Jaccard index (Quartile ranges are reported in Table 3). **A.** 1994−1996. **B.** 2004−2006. **C.** 2014−2016. **D.** 2014−2016 HCP.

Fig 4 illustrates the international scientific collaborations between cities by quarters of the Jaccard index. A clear observation is the increase of collaboration links between Western European cities and Northern American and Asian cities from 1994−1996 to 2004−2006 (Fig 4A and 4B). This observation is in line with previous findings on the rapid globalization of science [94, 95]. Second, in the case of the Q1 (the strongest) collaboration links, between 1994−1996 and 2004−2006, the Western European−United States links became dominant among the strongest city−city links. By the period of 2014−2016, the strongest inter-city links became more diffused across continents, with an emerging presence of African, Latin American, and Middle Eastern cities due to which the ranks of some links among Northern America, Europe, and East Asia lowered (Fig 4C). In contrast, high-impact collaborations across Northern America, Europe, and Asia were ranked higher than average collaborations, whereas collaboration between Europe and the emergent cities in Latin America and Africa did not lose importance compared to average collaborations (Fig 4D).

To examine more closely the emergence of cities in the strongest international collaborations, we aggregate the number of links by continents and macro-regions and report the ratios of these aggregates in Table 4. In each period, the share of Western Europe was highest, which is not particularly surprising because the highest number of cities in the network are from Western Europe (i.e., 92 cities, the 37.55 percent of all cities in the dataset). However, the dominance of Western Europe is even larger in Q1 collaboration links, signaling that international collaboration is a European phenomenon, which is partly due to the large number of cities distributed across many countries in Europe. However, the dynamics of the network implies that this dominance is not automatic. In the first period, EU cities had 3,469 links that decreased to 3,249 by 2004−2006 but rose again to 3,675 by 2014−2016. EU cities (both Eastern and Western) have even a larger share among the strongest international collaborations that have high Jaccard values as well (Q1).

**Table 4:** International collaboration links of cities by macro-regions.

	Ratio of collaboration links by macro-regions (%)				Ratio of Q1 collaboration links by macro-regions (%)			
	1994–1996	2004–2006	2014–2016	2014–2016, HCPs	1994–1996	2004–2006	2014–2016	2014–2016, HCPs
Africa	0.050	0.067	2.317	2.033	0.000	0.000	3.267	2.733
Asia	5.917	8.267	2.583	5.850	0.867	2.600	1.467	3.867
Australia	0.517	1.033	1.333	1.583	0.000	0.400	0.000	0.267
Eastern Europe	7.533	5.683	10.417	11.733	10.067	4.267	13.533	20.400
Latin America	1.600	1.700	3.917	6.383	1.867	0.667	2.600	7.667
Middle East	1.783	0.467	2.900	5.033	1.867	0.000	1.600	6.133
Northern America	24.467	27.817	16.933	15.500	8.333	26.933	7.867	8.067
Western Europe	58.133	54.967	59.600	51.883	77.133	65.133	69.667	50.867
**TOTAL**	**100.000**	**100.000**	**100.000**	**100.000**	**100.000**	**100.000**	**100.000**	**100.000**
European Union*	*57*.*817*	*54*.*150*	*61*.*250*	*54*.*683*	*78*.*933*	*63*.*533*	*71*.*400*	*57*.*267*

* In the case of the European Union, the community of 28 member states is considered, irrespective of which of the periods is examined

This dynamic for Northern American and Asian cities was the opposite over the three decades. Northern American cities had 1,468 links in 1994−1996, 1,669 links in 2004−2006, and 1,016 links in 2014−2016. Asian cities have increased their links from 1994−1996 to 2004−2006 in China (82→137), Japan (170→191), and South Korea (55→119). However, and irrespective of the increasing participation of East Asian cities in international scientific collaborations, most of these links have low Jaccard values (Fig 4B). More surprisingly, there were only five links of Chinese cities among the strongest international collaborations in 2014−2016, none of which had a high Jaccard. This finding is due to the fact that recently, Chinese cities have experienced a substantially more robust increase in their total publication output as compared to the number of their internationally co-authored publications (i.e., in the case of Chinese cities, the value of the Jaccard index has become smaller over time) [41, 49, 96]. In contrast to the Northern American and Asian trends, the ratio of African, Latin American, and Middle Eastern links has risen by 2014−2016.

The European Union dominates the international collaboration of cities partly due to the research policy of the Community. In the beginning of the 2000s, the European Research Area (ERA) was established, which was motivated by efficiency gains of developing a pan-European science base instead of coordinating national efforts in order to avoid lagging behind other major global players and create a “new European-level funding mechanism to support the very best research carried out at the frontiers of knowledge” [97–100]. Since the launch of the Sixth Framework Programme (2002−2006), the funding instrument to support and foster the construction of the ERA, the key goals of research funding are deepening the research collaboration between institutions located in the Member States [101, 102]. However, critics argue that the distance decay of research collaboration in Europe is a sign that ERA is not functioning optimally [102].

Comparing the participation of macro-regions in high-impact collaborations with participation in lower-impact collaborations, we observe that participation of the European Union, Western Europe in particular, and Northern America underperform, whereas Latin America, the Middle East, and Eastern Europe account for high ratios, particularly when links of the highest Jaccard values are only considered. Certainly, the Q1 set of links is biased towards cities that have few links, which produces an even stronger underrepresentation of Northern America and Western Europe, an important artifact of the analysis to keep in mind.

In the following section, we build on the findings in Section 3.2 and attempt to interpret the distribution of high-impact international collaboration as a result of big science and large-scale research projects because the highest proportion of high-impact international collaborations are materialized in fields where big science and other highly complex research projects are increasingly dominant. We offer three interpretations of high-impact distributions.

First, recently, core regions (i.e., the United States and the European Union) tend to establish more intensive research collaboration with developing countries involving researchers from the latter ones to participate in big science and other highly complex research projects [92, 103–105]. This collaboration is important for core regions because some infectious diseases (e.g., Ebola and Malaria), geological phenomenon, and environmental problems can be best studied in developing countries, which requires the participation of local experts and researchers [104]. In addition, the involvement of developing countries in collaborative projects can serve to improve international political stability as well as transfer vital skills and technologies to other parts of the world [105].

Second, the ERA is an effective tool for producing strong collaboration links for high-impact output. The relatively large ratio of Eastern Europe in such projects is a sign of this ability. Further, findings presented in Section 3.1 suggest that most intensive high-impact collaboration occurs across cities that are less than 4,000 kilometers away from each other on average, suggesting that there are many such links across European cities.

Third, Northern America remains relatively isolated from international high-impact science. US cities are high-impact producers themselves, which decreases the relative importance of collaborations. In addition, due to the fact that the United States has the largest science system in the world with many actors (e.g., universities, research institutes, and corporate labs) within that system, the ratio of the national collaboration is remarkably high [106]. In the case of big science, even the traditionally strong connections between the United States and Western European cities [107, 108] become less cooperative, and the two large science systems tend to carry out such large-scale research projects in parallel. For example, two neuroscience initiatives were launched in 2013 with almost equal budgets: the BRAIN Initiative of the US National Institutes of Health and the Human Brain Project, the flagship project of the European Commission [109, 110]. Similar parallel investments occurred in the construction of next-generation neutron sources that an OECD report in 1998 strongly recommended to carry out in America, Europe, and Asia [111]. In 2006, the United States put the SNS, a pulsed spallation neutron source into operation in the Oak Ridge National Laboratory, and in 2009 Japan followed it with the Japan Proton Accelerator Research Centre in Tokai. In the European Union, the Lund-based European Spallation Source (ESS) is currently under construction and is intended to be the world's most powerful next-generation neutron source.

As per the official statements coming from representatives located on both sides, the United States and the European Union are committed to maintaining strong trans-Atlantic scientific cooperation [112, 113]. That is, the question remains: What is the reason for the United States and the European Union each intending to run big science projects with similar scientific goals in parallel and not in cooperation if the collaboration is supported by (science) politicians? In fact, if digging more deeply, we can find evidence of sharp competition between the United States and the European Union. Taking beam physics as an example, Kaiserfeld adds [114] that “when European expressed hopes that the new spallation sources in Japan and the US might also accommodate the need for neutrons among European scientists, representatives from the SNS and the US Department of Energy ‘firmly contradicted’ them”. As a matter of fact, “competition was the word now used to inject courage into the struggling ESS project—not competition between European countries, but between Europe and other countries.” It is assumed that such competition exists in other fields as well, and it could be one reason why there is only weak relative collaboration between US and EU cities.

## 4. Discussion and conclusions

In this paper, we demonstrated the effect of geographical proximity on the relative strength of international scientific collaboration between cities over time. Our research was centered on three research questions:

1) Due to multiple factors, but the rapid development of information and communication technologies in the first place, the intensity of international scientific collaboration has significantly increased recently; yet, geographical proximity has still remained a restrictive factor for the actors involved in scientific cooperation.

First, we found that, in the past 30 years, particularly since the mid-2000s, the relative strength of international scientific collaboration in general had increased to a significant extent; that is, as compared to their total publication outputs, cities tend to produce a growing number of internationally co-authored publications. Second, the mean geographical distance of international scientific collaborations between cities, even in the case of the relative strongest collaborations, has become substantially higher over time. This finding suggests that recently, cities have been constructing scientific collaborations with their peers even if they are located at an increased geographical distance from each other. In addition, in the past two decades, a growing number of cities from developing countries has joined the scientific realm created by core countries, subsequently contributing to an increase in the mean geographical distance of collaborations. Yet, irrespective of which time period is observed, the geographical proximity still impacts the collaborations between cities. That is, by acknowledging the overall increase in the mean geographical distance of international scientific collaborations, we experienced that relatively strong collaborations still required smaller geographical distances. More precisely: the relatively strongest collaborations were generally created between cities located in neighboring countries.

2) The supranational policies fostering international scientific collaborations, particularly in the case of the European Union, help lessen the restrictive effect of geographical proximity.

Until the mid-2000s, a growing number of cities across the world, but particularly those located in Asia and Eastern Europe, had started to join the international arena of science and construct relatively strong collaborations with other cities. Furthermore, Northern American cities also became more collaborative in terms of the number of internationally co-authored papers. During the mid-1990s to the mid-2000s, it was the European Union (i.e., the totality of the old and new members of the EU-28) that experienced a decreasing number of cities in international collaborations. Then, since the mid-2000s, radical changes have taken place: Cities from the European Union have occupied the vast majority of collaboration links, whereas the ratio of Northern American cities (US cities in the first place) in those collaboration links has almost been halved and the participation ratio of Asian cities has become rather insignificant.

We propose two major reasons behind these changes. First, the European Union’s largest single enlargement in terms of people and the number of countries took place in 2004, when eight Central and Eastern European (CEE) and two Mediterranean countries joined the Community. This was followed by the accession of two more CEE countries in 2007. By the mid-2010s, the European Union became the political and economic integration of 28 member states. After the accession of CEE countries to the European Union, they were able to receive support from the EU’s Structural Funds and the Cohesion Fund, allowing those countries to improve the infrastructure of their national science system and pay additional money to researchers. Second, since the beginning of the 2000s, by the establishment of the ERA, the Community has made significant efforts to reduce the fragmentation of the European research landscape, and the isolation and compartmentalization of national research systems [115]. In addition, in 2009, the legal framework for the European Research Infrastructure Consortium (ERIC) was put in force to facilitate the establishment and operation of research infrastructure with European interest. Under the umbrella of the ERIC, a number of large-scale multi-Member States research infrastructure projects have been implemented, one of which is the ESS in Lund.

Due to these developments, international scientific collaborations between cities located in the European Union have been given significant impetus. We also found, however, that even in the case of the European Union, the geographical proximity still affects the relative strength of collaborations. This observation suggests that irrespective of the positive impact of supranational policies and the number of financial incentives, cities mostly tend to collaborate with their peers located in neighboring countries.

3) In the case of international scientific collaborations resulting in excellent papers, due to the high complexity of research projects, the restrictive effect of geographical proximity will be less significant.

To answer this question, in the period of 2014−2016, we compared the relative strength of international scientific collaborations and the geographical pattern of those collaborations in the case of all papers and highly cited papers (HCPs). The results demonstrate that, based on a disciplinary analysis of cities’ outputs, the majority of HCPs are the outcomes of big science and other large-scale research projects carried out in the fields of life sciences and physical science. These projects have common features in that they require the most cutting-edge research infrastructure and the cooperation of huge researcher teams, being sometimes constituted by hundreds or thousands of individuals. In addition, due to the extremely high costs generally characterizing big science projects, they might require co-funding of multiple nations. Considering these factors, it is not surprising that in the case of HCPs, the intensity of international collaborations in terms of output vs. co-produced papers ratio is substantially higher than in the case of all papers. Another observation is that HCP collaborations are less constrained by the effect of geographical proximity; that is, cities construct relatively strong collaborations with their peers located at a significantly increased physical distance from them. Yet, from a threshold interval of 4,000−5,000 kilometers, the intensity of HCP collaborations begins to lessen. These facts suggest that the United States and the European Union (even Japan in some cases), the global leaders in science, tend to carry out big science projects on their own and not in cooperation. Now, “big science” can be labelled by such terms as prestige [116], nationalism [117], and competition [114]. One exception is considered the quite successful Human Genome Project (HGP), which was carried out in collaboration with major scientific actors (i.e., the United States, some member states of the European Union, and China and Japan). The implementation of the HGP demonstrates the manner in which big science should be approached to surmount the challenges posed by the new coronavirus (COVID-19) [118, 119].

In addition, by examining the impact of distance on big science collaborations, a further research question emerges. Big science projects can be carried out either in a research lab that has a specific geographical location (e.g., CERN and the Oak Ridge National Laboratory), or by international research teams of whom members are located geographically separated (e.g., the HGP and the Human Brain Project). Gibbons et al. [120] suggest that due to the development of such platforms as the Internet, we are now experiencing the emergence of a socially distributed knowledge production system. Follow-up research should focus on investigating the differences in the evolution of geographically concentrated big science and distributed knowledge production because these modes have varying effects on the distance of collaborations.

## Supporting information

S1 Fig Mean Jaccard index by distance. (DOCX)

S1 Table Correlation between inter-city link strength. (DOCX)
