# Supplementary figures and images for: Exploring the changing geographical pattern of international scientific collaborations through the prism of cities
Source: PLoS One. 2020 Nov 16;15(11):e0242468. doi: 10.1371/journal.pone.0242468 (PMC7668612; doi:10.1371/journal.pone.0242468)

**S1 Fig. Mean Jaccard index by distance.**


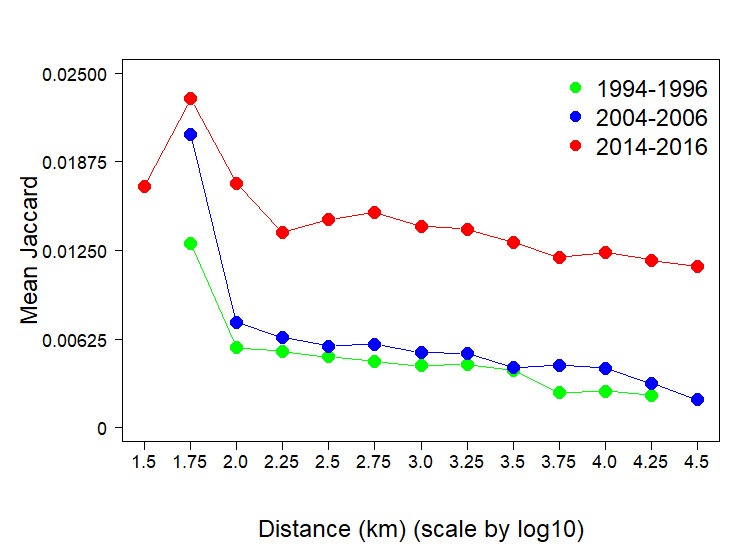


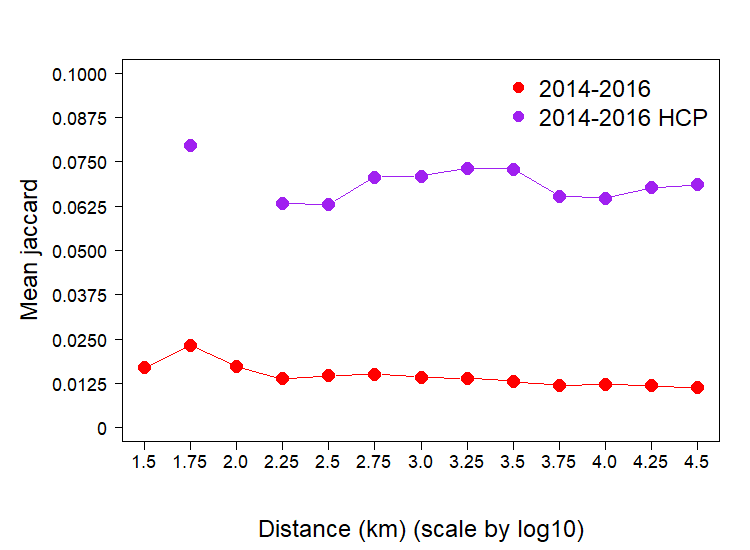

Supplement: S1 Fig — (DOCX) [file pone.0242468.s001.docx]
