# Supplementary material for: Exploring the changing geographical pattern of international scientific collaborations through the prism of cities
Source: PLoS One. 2020 Nov 16;15(11):e0242468. doi: 10.1371/journal.pone.0242468 (PMC7668612; doi:10.1371/journal.pone.0242468)
Supplement: S1 Table — (DOCX) [file pone.0242468.s002.docx]

**S1 Table. Correlation between inter-city link strength.**

|  |  | **1** | **2** | **3** | **4** | **5** | **6** | **7** |
| --- | --- | --- | --- | --- | --- | --- | --- | --- |
| 1 | *C_94−96_* | 1 |  |  |  |  |  |  |
| 2 | *J_94−96_* | 0.47 | 1 |  |  |  |  |  |
| 3 | *C_06−06_* | 0.66 | 0.01 | 1 |  |  |  |  |
| 4 | *J_04−06_* | 0.34 | 0.58 | 0.42 | 1 |  |  |  |
| 5 | *C_14−16_* | 0.69 | -0.17 | 0.83 | -0.01 | 1 |  |  |
| 6 | *J_14−16_* | 0.35 | 0.48 | 0.23 | 0.50 | 0.31 | 1 |  |
| 7 | *C_14−16HCP_* | 0.59 | -0.23 | 0.74 | -0.03 | 0.93 | 0.02 | 1 |
| 8 | *J_14−16HCP_* | 0.25 | 0.26 | 0.12 | 0.21 | 0.15 | 0.47 | 0.02 |
